# Interaction Energy Analysis of Monovalent Inorganic Anions in Bulk Water Versus Air/Water Interface

**DOI:** 10.3390/molecules26216719

**Published:** 2021-11-06

**Authors:** John M. Herbert, Suranjan K. Paul

**Affiliations:** Department of Chemistry & Biochemistry, The Ohio State University, Columbus, OH 43210, USA; paul.926@osu.edu

**Keywords:** air–water interface, Hofmeister series, hydrogen bonding, charge transfer, symmetry-adapted perturbation theory, noncovalent interactions

## Abstract

Soft anions exhibit surface activity at the air/water interface that can be probed using surface-sensitive vibrational spectroscopy, but the structural implications of this surface activity remain a matter of debate. Here, we examine the nature of anion–water interactions at the air/water interface using a combination of molecular dynamics simulations and quantum-mechanical energy decomposition analysis based on symmetry-adapted perturbation theory. Results are presented for a set of monovalent anions, including Cl^−^, Br^−^, I^−^, CN^−^, OCN^−^, SCN^−^, ${\mathrm{NO}}_{2}^{-}$, ${\mathrm{NO}}_{3}^{-}$, and ${\mathrm{ClO}}_{n}^{-}$ (n=1,2,3,4), several of which are archetypal examples of surface-active species. In all cases, we find that average anion–water interaction energies are systematically larger in bulk water although the difference (with respect to the same quantity computed in the interfacial environment) is well within the magnitude of the instantaneous fluctuations. Specifically for the surface-active species Br^−^(aq), I^−^(aq), ${\mathrm{ClO}}_{4}^{-}$(aq), and SCN^−^(aq), and also for ClO^−^(aq), the charge-transfer (CT) energy is found to be larger at the interface than it is in bulk water, by an amount that is greater than the standard deviation of the fluctuations. The Cl^−^(aq) ion has a slightly larger CT energy at the interface, but ${\mathrm{NO}}_{3}^{-}$(aq) does not; these two species are borderline cases where consensus is lacking regarding their surface activity. However, CT stabilization amounts to <20% of the total induction energy for each of the ions considered here, and CT-free polarization energies are systematically larger in bulk water in all cases. As such, the role of these effects in the surface activity of soft anions remains unclear. This analysis complements our recent work suggesting that the short-range solvation structure around these ions is scarcely different at the air/water interface from what it is in bulk water. Together, these observations suggest that changes in first-shell hydration structure around soft anions cannot explain observed surface activities.

## 1. Introduction

One of the earliest results of surface-sensitive vibrational sum-frequency generation (VSFG) experiments [1,2] was the observation that soft anions impact the vibrational lineshape in the O–H stretching region, but that hard anions do not [3,4,5,6]. The term “soft” is chosen carefully here, as an alternative to “polarizable”; it can be roughly interpreted as monovalent and polarizable, equivalent to having a low surface charge density [7], and such ions are sometimes called “chaotropic” [8]. Although the surface activity of certain anions is often discussed in terms of polarizability [9,10,11,12,13,14,15,16,17], it should be noted that polyvalent anions such as ${\mathrm{SO}}_{4}^{2-}$(aq) are quite polarizable [18] but the presence of polyvalent anions in solution does not affect the O–H lineshape measured in VSFG experiments [19]. Molecular dynamics (MD) simulations suggest that hard anions, including polyvalent species but also fluoride, are repelled from the air/water interface [20,21].

The nature of the surface activity exhibited by soft anions remains a matter of debate. Whereas continuum electrostatics predicts that all ions are repelled from the air/water interface [13], a first wave of MD simulations using polarizable force fields suggested that soft anions are not only present at the interface but in fact partition preferentially there [9,13,20,22]. More recent work suggests that these concentration enhancements were exaggerated by the force fields in use at the time [23,24,25,26,27,28,29], which aligns with the interpretation of some of the early experiments [3]. According to this point of view, surface activity may simply reflect the absence of depletion of soft anions at the interface [30,31], rather than a concentration enhancement.

To this debate, the present authors have recently added the observation that the first-shell hydration structure around soft anions is hardly different at the air/water interface as compared to that in bulk water [7]. This observation comes from MD simulations using polarizable force fields, and such similarities had been noted previously in simulations of I^−^(aq) [32] and SCN^−^(aq) [33], in the latter case using *ab initio* MD. Iodide and thiocyanate are archetypal examples of ions that perturb the O–H lineshape in VSFG experiments [3,4,19,34,35]. Our work [7] considered a larger set of anions, and the structural similarities that we observe, including the number and orientation of the ion–water hydrogen bonds, suggest that the origins of anion-induced changes in the O–H vibrational lineshape must be rather subtle effects on water–water hydrogen bonds, perhaps due to ion-induced changes in local electric fields [36]. These observations need to be reconciled with the prevailing modern view that monovalent ions have little effect on the long-range hydrogen-bonding *dynamics* of liquid water [37], as measured by femtosecond vibrational pump–probe experiments [37,38,39,40], although the effects on the long-range hydrogen-bonding *structure* of water are less clear. Neutron diffraction experiments [41,42,43,44] and some MD simulations [45] do suggest that even monovalent ions alter the tetrahedral ordering of water beyond the first solvation shell, for solutions of NaOH(aq), HCl(aq), NaCl(aq), and KCl(aq). Pronounced structural changes have been documented in some cases involving polyvalent ions [46,47,48].

Our previous work [7] was limited to structural characterization of the ions in question, along with a detailed examination of their ionization energies in order to make contact with liquid microjet photoelectron spectroscopy [49]. The present work adds another dimension to this analysis as we compute anion–water interaction energies for the same set of anions: Cl^−^, Br^−^, I^−^, CN^−^, OCN^−^, SCN^−^, ${\mathrm{NO}}_{2}^{-}$, ${\mathrm{NO}}_{3}^{-}$, and ${\mathrm{ClO}}_{n}^{-}$ (n=1,2,3,4). Some of these are typical surface-active ions (e.g., Br^−^, I^−^, SCN^−^, and ${\mathrm{ClO}}_{4}^{-}$), whereas others (such as CN^−^, OCN^−^, and ${\mathrm{NO}}_{2}^{-}$) visit the interface much less frequently, according to the MD simulations [7], and are not classified as surface-active. Intermediate cases where the surface activity is weak, or where experimental consensus is lacking, include Cl^−^ and ${\mathrm{NO}}_{3}^{-}$ [19]. Amongst these ions, our simulations indicate that even the ones that are not considered surface active nevertheless spend enough time near the air/water interface that it is possible to assemble an interfacial data set for them. These cases offer a useful comparison to the canonical surface-active anions.

We present a detailed analysis of the (ensemble-averaged) interaction between each of these ions and its short-range hydration sphere, in both bulk water and at the air/water interface, using the quantum-chemical methods of symmetry-adapted perturbation theory (SAPT) [50,51,52,53]. The SAPT family of methods [50,51] is designed for accurate calculation of noncovalent interaction energies, as well as a physically-motivated energy decomposition analysis of those energies [51,52]. Of key interest will be whether the interfacial environment engenders any discernible changes in the ion–water interactions, relative to what is observed for the same ion in bulk water.

## 2. Computational Methods

### 2.1. Classical MD Simulations

MD simulations of the aforementioned ions in a periodic slab configuration were reported previously [7] and the same set of simulations is used here to obtain snapshots for interaction energy analysis. These simulations were performed under NVT conditions at T=298 K and a bulk density of 0.997 g/cm^3^. The size of the periodic simulation cell (31.3 Å × 31.3 Å × 156.7 Å) was previously shown to afford converged results [7]. The simulation data were subsequently partitioned into bulk and interfacial parts depending on the position of the ion relative to the Gibbs dividing surface (GDS) that we take to define the air/water interface. For the snapshots classified as “interfacial”, the ion’s center of mass lies no more than 3 Å below the GDS. Anything beyond this cutoff is considered to be a bulk water environment, as this interior region of the periodic slab affords properties that are essentially indistinguishable from results performed in an isotropic simulation that has no interface [7]. Simulations were performed using the AMOEBA force field for water [54], whose parameterization includes some of the ions in question, such as the halides [55]. Parameters for the remaining ions were developed along similar lines [7], following an established protocol [56], and are included in the Supplementary Material. Energetic analyses with the AMOEBA force field were performed using Tinker software, v. 8 [57].

Following an equilibration period, snapshots were extracted that include two solvation shells around the ion, according to distance criteria described previously [7]. The number of water molecules varies from one snapshot to the next, with the average number $\langle N_{w}\rangle$ depending on both the size of the ion and how tightly hydrated it is. In bulk water, these averages range from $\langle N_{w}\rangle$ ≈ 28 for Cl^−^(aq) up to $\langle N_{w}\rangle$ ≈ 43–44 for Br^−^(aq) and I^−^(aq), with $\langle N_{w}\rangle$ = 35–37 for the remaining ions. The interfacial snapshots contain fewer water molecules, on average, as the water density is reduced in the interfacial region. In the analysis that follows, we consider interaction energies ($E_{\mathrm{int}}$) between the ion and its first two hydration shells. The quantity $E_{\mathrm{int}}$ is intensive with respect to system size and this insulates our analysis against the step-to-step fluctuations in the number of water molecules that are included in these calculations. Ensemble averages reported below represent 51 snapshots for each ion in bulk water, as well as 51 snapshots for each ion at the air/water interface, with individual snapshots separated by 10 ps in time. These ensembles were taken from our previous work [7], and coordinate files for these data sets are provided in the Supplementary Material. The bulk ensemble represented by these 51 snapshots affords statistical distributions that are indistinguishable from results obtained from an isotropic simulation, and the interfacial ensemble affords similar distributions regardless of whether the interface is defined by GDS−3 Å (as in the present work) versus GDS−1 Å or GDS−5 Å [7].

### 2.2. Symmetry-Adapted Perturbation Theory

Quantum-mechanical values of $E_{\mathrm{int}}$ were computed using SAPT based on Hartree-Fock (HF) wave functions for the monomers and second-order perturbation theory for the intermolecular Coulomb operators. This approach is usually called SAPT0 [51,58,59] and is closely related to second-order Møller-Plesset perturbation theory (MP2). However, because second-order dispersion is far from quantitative [50,51,60], we replace it in these calculations with a many-body dispersion (MBD) model [51,61,62], in what we have termed a “hybrid” or “extended” form of SAPT [51]. This hybrid method will be designated as SAPT0 + MBD. At this level of theory, results for small-molecule data sets suggest that errors in $E_{\mathrm{int}}$ are within ∼1 kcal/mol of the best-available benchmarks [59,62], provided that adequate basis sets are employed [59,63]. All electronic structure calculations were performed using Q-Chem software, v. 5.4 [64].

The interaction energy computed using SAPT0 + MBD is naturally partitioned as [50,51]

(1) $$E_{\mathrm{int}}=E_{\mathrm{elst}}+E_{\mathrm{exch}}+E_{\mathrm{ind}}+E_{\mathrm{disp}}.$$

The energy components [51,65] include electrostatics ($E_{\mathrm{elst}}$), meaning the Coulomb interaction between isolated-monomer charge densities; exchange or Pauli repulsion ($E_{\mathrm{exch}}$), which is the penalty to antisymmetrize the isolated-monomer wave functions; induction ($E_{\mathrm{ind}}$), which includes both polarization and charge transfer (CT); and, finally, dispersion ($E_{\mathrm{disp}}$). In our approach,

(2) $$E_{\mathrm{elst}}\equiv E_{\mathrm{elst}}^{\left(1\right)}$$

and

(3) $$E_{\mathrm{exch}}\equiv E_{\mathrm{exch}}^{\left(1\right)}$$

are the first-order SAPT electrostatic and exchange energies, while $E_{\mathrm{disp}}$ is the dispersion energy computed using the MBD model [62]. The induction energy comes from second-order SAPT but warrants additional discussion, which we defer until Section 2.3.

**Figure 1:**
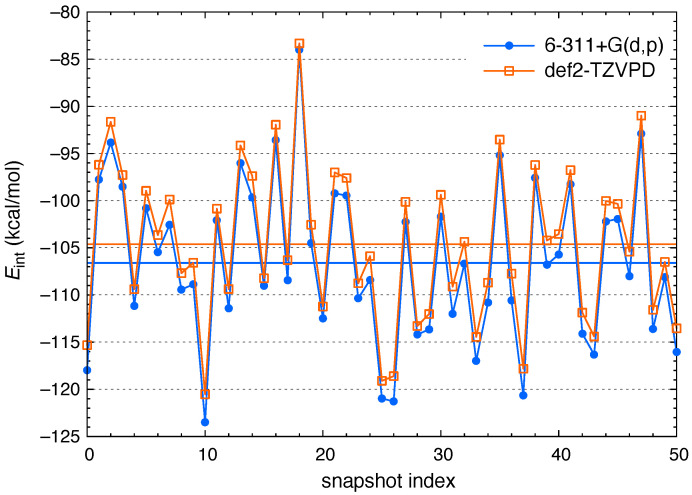
Total interaction energies for snapshots of Cl^−^(aq) in bulk water, computed at the SAPT0 + MBD level using two different basis sets. Solid horizontal lines show the ensemble-averaged values obtained using either basis set. These averages are $\langle E_{\mathrm{int}}\rangle =-106.6\pm 8.4$ kcal/mol for 6-311+G(d,p) and $\langle E_{\mathrm{int}}\rangle =-104.6\pm 8.2$ kcal/mol for def2-TZVPD, where the uncertainties represent one standard deviation.

Previous basis-set testing of SAPT0 + MBD reveals that polarized triple-*ζ* basis sets, augmented with diffuse functions, are both necessary and sufficient to obtain converged energetics [59,63]. This is a unique feature of our hybrid approach to SAPT [51], which replaces the very slow basis-set convergence of perturbative dispersion with a model (namely, MBD) that converges quickly [63]. Tests for Cl^−^(aq) in Figure 1 demonstrate that interaction energies computed using the 6-311+G(d,p) basis set agree with SAPT0 + MBD/def2-TZVPD values to within an average of 2.0 kcal/mol, in a total interaction energy that averages −106 kcal/mol. Relative to the more complete def2-TZVPD basis set, the Pople basis set systematically underestimates $E_{\mathrm{ind}}$ (by an average of 1.6 kcal/mol) and overestimates $E_{\mathrm{elst}}$ (by an average of 4.2 kcal/mol), whereas $E_{\mathrm{exch}}$ and $E_{\mathrm{disp}}$ are nearly identical in both basis sets.

More important than these relatively small differences is the fact that instantaneous values of $E_{\mathrm{int}}$ fluctuate from snapshot to snapshot in a similar way in either basis set. For these calculations, which involve Cl^−^(H_2_O)*_n_* with an average of n=28 water molecules, SAPT0 + MBD/6-311+G(d,p) calculations are 17× faster than the corresponding calculations with def2-TZVPD. (This speedup results largely from the absence of diffuse functions on hydrogen but also benefits from Q-Chem’s very efficient handling of sp shells in Pople basis sets.) In the present work, we are concerned with comparisons between bulk and interfacial behavior rather than absolute interaction energies, and the need for ensemble averaging requires high throughput. As such, 6-311+G(d,p) is used for all subsequent SAPT calculations.

Interaction energies defined in Equation (1) do not include relaxation of the monomer geometries, so $E_{\mathrm{int}}$ is an interaction energy in the “vertical” sense, not a binding energy or a solvation energy. In considering the ion–water clusters X^−^(H_2_O)*_n_* extracted from MD simulations, we treat the entire water cluster (H_2_O)*_n_* as a single monomer for the purpose of computing $E_{\mathrm{int}}$ and its components, then average over the ensemble of snapshots. Even so, the ensemble-averaged value $\langle E_{\mathrm{int}}\rangle$ corresponds to vertical removal of the ion. It includes the change in electronic polarization of the water molecules upon removal of the ion but does not include the orientational reorganization energy of the water to fill the void left behind by the ion.

Unless otherwise specified, all of the SAPT0 calculations reported herein use HF wave functions for the monomers. However, we will also report a few SAPT0(KS) calculations [51,59], in which Kohn-Sham (KS) molecular orbitals from density functional theory (DFT) are used in place of HF orbitals. These SAPT0(KS) calculations employ the long-range corrected (LRC) density functional LRC-*ω*PBE [66]. Previous work has emphasized the importance of using an asymptotically correct exchange potential in SAPT calculations [59,60,67,68], and this condition can be achieved in practice via monomer-specific tuning of the range-separation parameter (*ω*) in the LRC-*ω*PBE functional. Although “optimal tuning” of LRC functionals [69,70] is sometimes accomplished using the ionization energy (IE) theorem of DFT,

(4) $$\mathrm{IE}=-\varepsilon_{\mathrm{HOMO}},$$

a more robust procedure in the present context is the “global density-dependent” (GDD) or “$\omega_{\mathrm{GDD}}$” procedure [59,60,68]. This approach, which adjusts *ω* based on the size of the exchange hole, mitigates the strong dependence on system size that is observed when using IE tuning [59], which might otherwise be a problem when studying water clusters of varying size [71]. For water, we use $\omega =0.277\;a_{0}^{-1}$, which represents an average over several cluster geometries. For the ions, we tune *ω* individually at the optimized gas-phase geometry of each, resulting in a range of values from $\omega =0.248\;a_{0}^{-1}$ for iodide and $\omega =0.261\;a_{0}^{-1}$ for bromide, where the tails of the anion’s density are most diffuse, up to $\omega =0.398\;a_{0}^{-1}$ for cyanate and $\omega =0.405\;a_{0}^{-1}$ for cyanide, where the density is most compact. (Note that LRC functionals switch from semilocal exchange to HF exchange on a length scale of ∼1/*ω*.)

In previous work, we have often used self-consistent charge embedding of the SCF monomer wave functions as a means to incorporate many-body polarization effects into a pairwise SAPT calculation, albeit implicitly [50,72,73,74,75]. However, the present study does not make use of any charge embedding, and instead the X^−^(H_2_O)*_n_* system is treated as a dimer with (H_2_O)*_n_* as one monomer. In principle, charge embedding could be used to describe these clusters more efficiently as (n+1)-body systems, but we have chosen not to do so here. The dimer approach makes the SAPT interaction energies more directly comparable to those obtained using the AMOEBA force field.

### 2.3. Polarization and Charge Transfer

In our calculations, the induction term in Equation (1) is defined as

(5) $$E_{\mathrm{ind}}=E_{\mathrm{ind}}^{\left(2\right)}+E_{\mathrm{exch}-\mathrm{ind}}^{\left(2\right)}+\delta E_{\mathrm{HF}}\;.$$

The first two terms are the second-order (SAPT0) induction and exchange-induction energies, and

(6) $$\delta E_{\mathrm{HF}}=\Delta E_{\mathrm{int}}^{\mathrm{HF}}-\left(E_{\mathrm{elst}}^{\left(1\right)}+E_{\mathrm{exch}}^{\left(1\right)}+E_{\mathrm{ind},\mathrm{resp}}^{\left(2\right)}+E_{\mathrm{exch}-\mathrm{ind},\mathrm{resp}}^{\left(2\right)}\right)$$

is the so-called “*δ*HF” correction [51]. It uses a counterpoise-corrected, supramolecular HF interaction energy ($\Delta E_{\mathrm{int}}^{\mathrm{HF}}$) to correct the SAPT0 interaction energy for induction effects beyond second order in perturbation theory, which is crucial for the accurate description of hydrogen bonds [51,59]. See reference [76] for a definition of the second-order response (“resp”) energies that appear in Equation (6).

As defined in SAPT, the induction energy in Equation (5) contains both polarization and CT,

(7) $$E_{\mathrm{ind}}=E_{\mathrm{pol}}+E_{\mathrm{CT}},$$

for reasons that are discussed in reference [77]. In the analysis of hydrogen bonding it is often of interest to separate these effects but that separation has historically been considered problematic. The dilemma is not limited to SAPT and many schemes for separating polarization from CT exhibit strong dependence on the choice of basis set [77]. To accomplish the separation in Equation (7) in a robust way that converges rapidly with respect to basis set, we use the machinery of a charge-constrained self-consistent field (SCF) calculation [78] to define a CT-free reference state. Here, the monomers are allowed to polarize one another but their charge densities are constrained to integrate to integer numbers of electrons. Because the SCF procedure is variational, lifting of this constraint necessarily lowers the energy (to that of the fully-relaxed SCF solution), and this energy lowering is taken to define $E_{\mathrm{CT}}$. The CT energy thus obtained is then subtracted from the SAPT induction energy to obtain the CT-free polarization energy, $E_{\mathrm{pol}}=E_{\mathrm{ind}}-E_{\mathrm{CT}}$ [77,79,80,81]. CT energies defined in this way are very nearly converged already in double-*ζ* basis sets [77]. This approach has previously been used to demonstrate that $E_{\mathrm{CT}}$ furnishes a driving force for formation of quasi-linear hydrogen bonds in binary halide–water complexes [65,81].

Implementation of the charge-constrained SCF procedure requires a method to count electrons, and Becke’s multicenter partition scheme [82] is commonly used for this purpose [78]. Becke’s approach first divides space into Voronoi cells [83], which are regions of space that are closest to a particular nucleus, and then applies a smoothing function at the boundaries of these polyhedra. Alternatively, and specifically for the purpose of defining a CT-free reference state in order to affect the partition suggested in Equation (7), a counting procedure based on fragment-based Hirshfeld (FBH) weighting has also been suggested [79,81]. In the latter approach, the number of electrons contained in fragment *A* is defined as

(8) $$N_{A}=\int w_{A}\left(r\right)\;\rho \left(r\right)\;dr,$$

where ρ(r) is the supramolecular electron density, which is integrated subject to a weighting function $w_{A}\left(r\right)$. That function is defined as

(9) $$w_{A}\left(r\right)=\frac{\rho_{A}^{0}\left(r\right)}{\sum_{B}\rho_{B}^{0}\left(r\right)},$$

where $\rho_{X}^{0}\left(r\right)$ is the charge density of isolated fragment *X*. The denominator in Equation (9) is thus a superposition of isolated-fragment densities.

The Becke scheme can also be conceptualized as a form of Equation (8) in which $w_{A}\left(r\right)$ is a smoothed version of a Heaviside step function, which switches rapidly between $w_{A}\left(r\right)=0$ and $w_{A}\left(r\right)=1$ at the boundaries of the Voronoi polyhedra. In practice, our implementation of Becke’s procedure uses “atomic size adjustments” [82], in which a set of empirical atomic radii [84] are used to adjust the boundaries of the Voronoi cells away from the midpoints of the internuclear vectors. As discussed below, this adjustment is crucial for systems with substantial size mismatch between nearby atoms.

**Figure 2:**
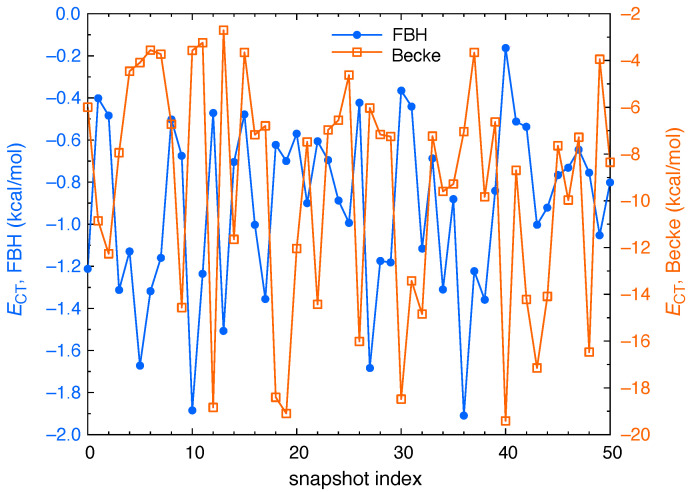
CT energies for snapshots of I^−^(aq) in bulk water, computed using a charge-constrained SCF procedure with the charge constraint defined using either fragment-based Hirshfeld (FBH) weighting (scale at left), or else Becke’s multicenter partitioning procedure (scale at right). Results using the Becke scheme include the “atomic size adjustments” that are described in reference [82], wherein Slater’s set of atomic radii [84] are used to adjust the boundaries of the Voronoi cells based on atomic size.

Even so, the FBH approach strikes us as the more reasonable one, especially where anions are involved, because Becke’s method depends only on the positions of the atoms (along with the empirical atomic radii), whereas the weight function defined in Equation (9) respects the diffuseness of the isolated anion’s wave function. In the present context, this almost inevitably means that the extent of anion → water CT is smaller when the FBH approach is used, because the tails of the X^−^ wave function cause a larger region of space to contribute to that fragment’s integrated number of electrons. As an example, Figure 2 presents $E_{\mathrm{CT}}$ computed using both Becke partition (with atomic size adjustments) and FBH weighting, for each snapshot of I^−^(aq) in bulk water. The results are considerably different depending on which method is used to count electrons, with the FBH approach compressing the CT energy into the interval $0>E_{\mathrm{CT}}>-2$ kcal/mol, whereas the Becke procedure affords values of $|E_{\mathrm{CT}}|$ as large as 20 kcal/mol. The latter value is comparable to the the average magnitude of the total SAPT0 induction energy, which is $\langle E_{\mathrm{ind}}\rangle =-22.3$ kcal/mol for I^−^(aq) in bulk water. (Note that energy components corresponding to attractive interactions are negative.)

**Figure 3:**
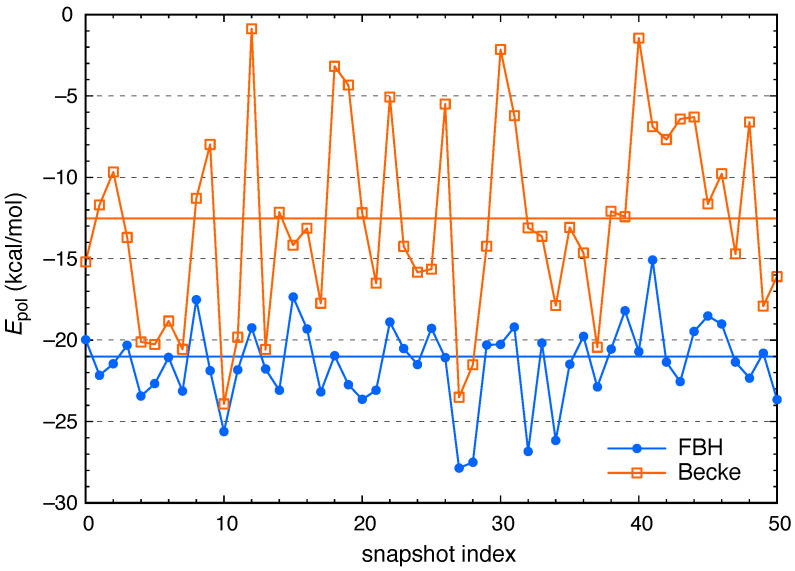
CT-free polarization energy ($E_{\mathrm{pol}}=E_{\mathrm{ind}}-E_{\mathrm{CT}}$) for snapshots of I^−^(aq) in bulk water. This quantity is obtained by removing $E_{\mathrm{CT}}$ from the total SAPT0 induction energy using either of two schemes (FBH weighting or Becke partition with atomic size adjustments) to integrate the charge constraint that defines the CT-free reference state. Solid horizontal lines show the ensemble-averaged values, which are $\langle E_{\mathrm{pol}}\rangle =-21.0\pm 3.9$ kcal/mol (FBH) and $\langle E_{\mathrm{pol}}\rangle =-12.6\pm 6.1$ kcal/mol (Becke). Uncertainties represent one standard deviation.

Figure 3 shows the polarization energy ($E_{\mathrm{pol}}=E_{\mathrm{ind}}-E_{\mathrm{CT}}$) that is obtained using either the Becke or the FBH weighting function to define the charge constraint. (Both definitions of $E_{\mathrm{pol}}$ start from the same SAPT0 induction energy, $E_{\mathrm{ind}}$.) It is apparent that the two definitions afford step-to-step fluctuations that do not seem to correlate with one another. In the Becke definition, the size and shape of the Voronoi cell that contains iodide is sensitive to the instantaneous values of all iodide–water distances in the first solvation shell, whereas the FBH definition uses a spherically-symmetric charge density for the isolated anion in order to define the charge constraint. The latter definition proves to be less sensitive to fluctuations in the atomic coordinates, although it remains sensitive to the presence of hydrogen bonds [65,81].

**Figure 4:**
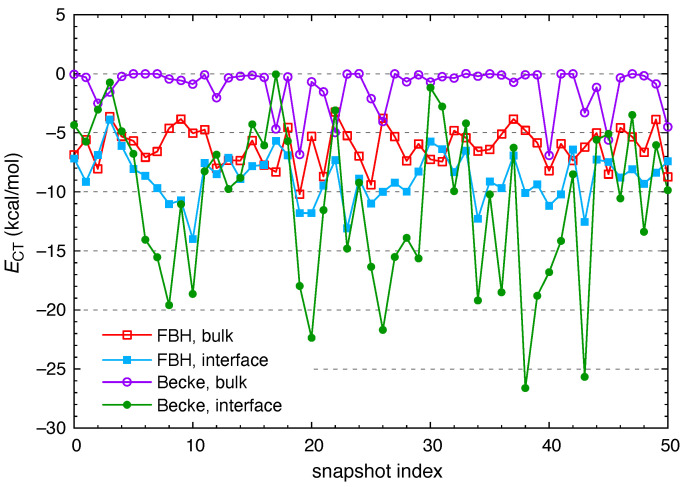
CT energies for ClO^−^(aq), computed using either Becke partition or else FBH weighting to define the CT-free reference state, and considering both the bulk and interfacial data sets.

For I^−^(aq), the CT-free reference state defined using Becke partition consistently results in CT energies that are larger in magnitude as compared to the FBH scheme: $|E_{\mathrm{CT}}\left(\mathrm{Becke}\right)|>\;|E_{\mathrm{CT}}\left(\mathrm{FBH}\right)|$. This is evident from the rather different energy scales in Figure 2, but the situation is not the same for all of the anions examined here. As a second example we consider ClO^−^(aq), which exhibits the largest values of $|E_{\mathrm{CT}}|$ amongst the anions in our data set, at least when the FBH definition is used. Figure 4 considers both definitions and examines how $E_{\mathrm{CT}}$ fluctuates from snapshot to snapshot. Becke’s partition predicts very little CT for ClO^−^(aq) in bulk water ($\langle E_{\mathrm{CT}}\rangle =-1.2$ kcal/mol), whereas the FBH definition results in an average value $\langle E_{\mathrm{CT}}\rangle =-6.2$ kcal/mol. In either case, $E_{\mathrm{CT}}$ is consistently larger for the interfacial snapshots.

We will use the FBH definition of $E_{\mathrm{CT}}$ for the remainder of this work. Our main interest lies in understanding how various energy components compare when the ion is in a bulk versus an interfacial environment, but the magnitude of $E_{\mathrm{CT}}$ can depend strongly on the method that is used to count electrons, as noted above. This observation suggests that in other applications of constrained DFT [78], which is the more common form of charge-constrained SCF calculation (in contrast to the constrained HF calculations employed here), the results should be checked carefully to ensure that conclusions are robust with respect to the details of how the constraints are implemented.

The SG-3 quadrature grid [85] is used to integrate the SCF constraint equations as well as Equation (8). As a technical aside, we note that the atomic size adjustments mentioned above are crucial in order to obtain results that are even remotely sensible when Becke partition is used to implement the charge constraint. However, the original implementation of constrained DFT in the Q-Chem program did not include these corrections [86], which were added recently for the purpose of SAPT-based CT analysis [81]. Absent these corrections, the Voronoi cell boundaries are placed at midpoints of the internuclear vectors, which affords unreasonable results in cases where neighboring atoms have very different size. This includes covalent bonds to hydrogen, where the midpoint definition causes too much density to be assigned to the smaller hydrogen atom, often leading to a negative charge assigned to hydrogen [81]. In the present work, neglecting the atomic size corrections leads to a significant fraction of the iodide’s charge being assigned to first-shell water molecules, resulting in completely unrealistic CT energies whose magnitudes exceed the total SAPT0 induction energy. In our view, constrained DFT based on Becke partition should not be used without the atomic size corrections.

## 3. Results and Discussion

**Figure 5:**
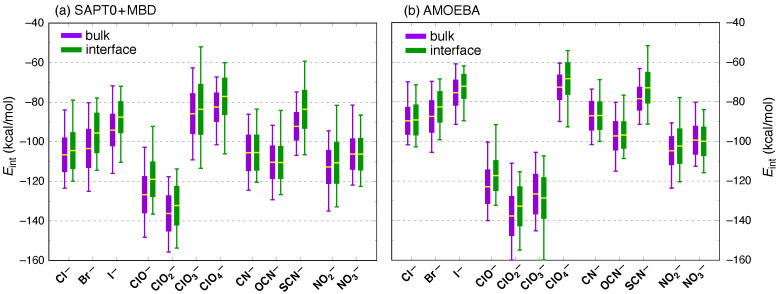
Total ion–water interaction energies between various anions and their first two hydration shells, computed using (**a**) quantum chemistry at the level of SAPT0 + MBD/6-311+G(d,p), versus (**b**) the AMOEBA force field that was used to obtain the structures. Side-by-side box and whisker plots are shown for each ion in bulk and interfacial environments. Box plots show the mean value $\langle E_{\mathrm{int}}\rangle$ (yellow bar) and extend for one standard deviation in both directions. Whiskers indicate minimum and maximum values of $E_{\mathrm{int}}$.

Figure 5 presents ensemble-averaged interaction energies for the sets of X^−^(H_2_O)*_n_* structures that are considered here, where X^−^ is one of 12 monovalent inorganic anions. Two solvation shells of surrounding water are treated as a single monomer for the purpose of the SAPT calculations. Results are presented both at the quantum-mechanical SAPT0 + MBD/6-311+G(d,p) level (Figure 5a) and also using the AMOEBA force field (Figure 5b), where the latter is the same force field that was used for the simulations from which these X^−^(H_2_O)*_n_* structures were extracted. Bulk and interfacial data are averaged separately, with the criterion GDS−3 Å used to decide whether a particular snapshot represents a bulk or an interfacial solvation environment.

There are two interesting observations to be made from the interaction energy data in Figure 5. Foremost is the fact that differences between the bulk and interfacial mean values $\langle E_{\mathrm{int}}\rangle$ for a given ion are small compared to the fluctuations in the instantaneous value of $E_{\mathrm{int}}$. Bulk values of $\langle E_{\mathrm{int}}\rangle$ are systematically (slightly) larger in magnitude than interfacial values, except for CN^−^, OCN^−^, and ${\mathrm{NO}}_{3}^{-}$ where the averages are essentially identical in both environments. In all cases, the difference between bulk and interfacial average values is well within the standard deviation in either quantity; see the numerical values that are provided in Table 1. For the halides, modest reductions in $\langle E_{\mathrm{int}}\rangle$ at the interface (up to 7–8 kcal/mol for bromide and iodide) are consistent with results from classical MD simulations indicating that the average ion–water interaction is reduced, for all of the halides, as the ion moves towards the interface [21]. It should be noted that the simulations reported in reference [21] indicate that the enthalpic portion of the potential of mean force is more favorable for the heavier halides at the interface, as compared to its value in bulk water. As such, the rather subtle differences between ion–water interactions that are documented in our quantum-mechanical calculations are more than compensated by ion-induced changes in the water–water interactions [21]. This is consistent with our detailed structural analysis of the ions [7], which indicates very little change in the first-shell structure at the interface as compared to that in bulk water.

A second interesting observation is the generally strong correlation between classical (AMOEBA) and quantum-mechanical (SAPT) values of $E_{\mathrm{int}}$, even if the former are systematically smaller than the latter, e.g., by 15–19 kcal/mol for the halide ions. (Systematic differences are smaller for the other ions, except in the case of ${\mathrm{ClO}}_{3}^{-}$. The latter is discussed in detail below.) For the halide ions, we use AMOEBA parameters that were originally developed by Ponder and co-workers [55], and we note that the discrepancies between the force field and the quantum chemistry that are documented in Figure 5 are much larger than those reported previously for binary X^−^(H_2_O) complexes [55]. This underscores the importance of considering larger ion–water clusters, given the many-body nature of polarization in aqueous systems [87,88,89,90,91,92]. Simulation of the hydration free energy of chloride using AMOEBA results in an error of 11.9 kcal/mol with respect to experiment [55], assuming that the reference value is defined using the proton solvation energy of Tissandier et al. [93], which has since emerged as the consensus value [94,95,96]. In view of this, the systematic difference of 17 kcal/mol between AMOEBA and SAPT0 + MBD values of $\langle E_{\mathrm{int}}\rangle$ in bulk water (see Table 1) is not so dissimilar from previous results. Improvements to the AMOEBA force field for ions, using SAPT energy components as benchmark data, is a topic of contemporary interest [97,98,99].

The chlorate (${\mathrm{ClO}}_{3}^{-}$) ion represents the lone exception to an otherwise systematic correlation between classical and quantum-chemical interaction energies. This particular species is much more strongly solvated by AMOEBA ($\langle E_{\mathrm{int}}\rangle =-126.6\pm 9.9$ kcal/mol in bulk water) than it is by SAPT0 + MBD ($\langle E_{\mathrm{int}}\rangle =-85.8\pm 10.0$ kcal/mol). Considering the chlorine oxyanions as a group, the trend amongst the AMOEBA values of $\left|\langle E_{\mathrm{int}}\rangle \right|$ is ${\mathrm{ClO}}_{2}^{-}>{\mathrm{ClO}}_{3}^{-}≳{\mathrm{ClO}}^{-}\gg {\mathrm{ClO}}_{4}^{-}$. The fact that perchlorate (${\mathrm{ClO}}_{4}^{-}$) is an outlier is easy to rationalize in terms of its tetrahedral symmetry and vanishing dipole moment, but the trend amongst the other three chlorine oxyanions is more puzzling. Ensemble-averaged SAPT0 + MBD energy components for the four species ${\mathrm{ClO}}_{n}^{-}$(aq) are listed in Table 2, from which it may be seen that $\langle E_{\mathrm{int}}\rangle$, $\langle E_{\mathrm{elst}}\rangle$, and $\langle E_{\mathrm{ind}}\rangle$ all follow the same trend exhibited by the gas-phase dipole moments of the ions in question. However, this means that the trend amongst total interaction energies is different from that predicted by AMOEBA. Instead, from the quantum-mechanical calculations the trend (from strongly to weakly interacting) is ${\mathrm{ClO}}_{2}^{-}>{\mathrm{ClO}}^{-}\gg {\mathrm{ClO}}_{3}^{-}≳{\mathrm{ClO}}_{4}^{-}$.

**Figure 6:**
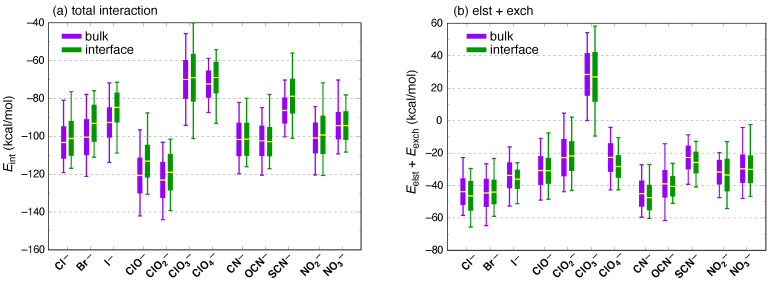
Box-and-whisker plots showing mean values, standard deviations, and extremal values of (**a**) the total interaction energy ($E_{\mathrm{int}}$) and (**b**) first-order electrostatics plus exchange ($E_{\mathrm{elst}+\mathrm{exch}}$) for ion–water clusters. These calculations were performed at the SAPT0(KS) + MBD/6-311+G(d,p) level of theory and therefore include intramolecular electron correlation effects. Panel (**a**) should be compared to Figure 5a, as the difference lies solely in whether HF or KS molecular orbitals are used within the SAPT0 formalism, and the vertical scales are the same in both figures. Similarly, panel (**b**) should be compared to Figure 7a although the vertical scales are slightly different.

In contrast to the AMOEBA results, the SAPT0 + MBD calculations afford similar ensemble-averaged interaction energies for both ${\mathrm{ClO}}_{3}^{-}$ and ${\mathrm{ClO}}_{4}^{-}$. Given that all of the chlorine oxyanions except for ${\mathrm{ClO}}_{4}^{-}$ have sizable dipole moments, the ${\mathrm{ClO}}_{3}^{-}$ interaction energy seems anomalously small when computed using SAPT0 + MBD. As a sanity check, we recomputed interaction energies for all of the ions using SAPT0(KS) + MBD, which includes intramolecular electron correlation. These results are plotted in Figure 6a, which should be compared to the corresponding SAPT0 + MBD results in Figure 5a. Total interaction energies at either level of theory are quite comparable, and in particular both methods exhibit the same (seemingly anomalous) trend amongst the ${\mathrm{ClO}}_{n}^{-}$ ions, which differs from the trend predicted by AMOEBA.

**Figure 7:**
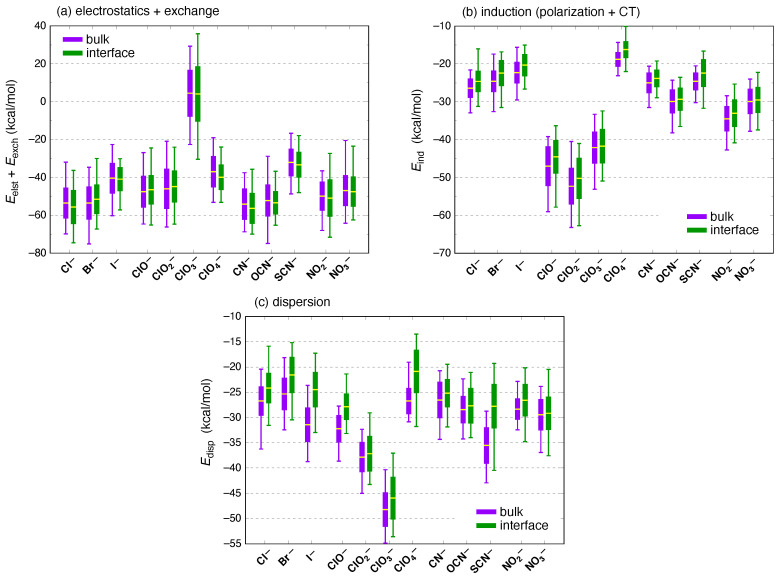
Box-and-whisker plots showing mean values, standard deviations, and extremal values of ion–water interaction energy components computed at the SAPT0 + MBD/6-311+G(d,p) level: (**a**) first-order electrostatics and exchange, $E_{\mathrm{elst}}+E_{\mathrm{exch}}$; (**b**) $E_{\mathrm{ind}}$ from Equation (5); and (**c**) $E_{\mathrm{disp}}$ from the MBD model. The sum of the energy components in panels (**a**–**c**) equals $E_{\mathrm{int}}$ in Figure 5a.

To investigate this further, we examine the SAPT0 + MBD energy components. These are plotted for each of the ions in Figure 7, again separating bulk and interfacial environments and ensemble-averaging over either data set. In considering the energy decomposition in Equation (1), we have opted to group first-order electrostatics and exchange together,

(10) $$E_{\mathrm{elst}+\mathrm{exch}}=E_{\mathrm{elst}}+E_{\mathrm{exch}},$$

because their sum approximates the electrostatic interaction between antisymmetrized monomer wave functions. This combination of “primitive” electrostatics ($E_{\mathrm{elst}}$, which is the Coulomb interaction between isolated-monomer charge densities) and Pauli repulsion ($E_{\mathrm{exch}}$) has proven to be easier to interpret for halide–water systems as compared to electrostatics alone [65,81], in part because $E_{\mathrm{elst}}$ and $E_{\mathrm{exch}}$ are the largest energy components (in magnitude) but often have opposite signs, such that their sum is more comparable in magnitude to the remaining energy components. An example can be found in the ensemble-averaged energy components for the ${\mathrm{ClO}}_{n}^{-}$(aq) species (Table 2), where the much less repulsive value of $\langle E_{\mathrm{exch}}\rangle$ for perchlorate at first seems at odds with the larger size of this ion. However, the reduced Pauli repulsion in this case is actually commensurate with a much less attractive value of $\langle E_{\mathrm{elst}}\rangle$, suggesting a hydration sphere that is not as tight around the ion as it is in the smaller (but electrostatically much more attractive) ClO^−^, ${\mathrm{ClO}}_{2}^{-}$, and ${\mathrm{ClO}}_{3}^{-}$ ions.

Statistical distributions of $E_{\mathrm{elst}+\mathrm{exch}}$ are shown in Figure 7a for all of the ions, and ${\mathrm{ClO}}_{3}^{-}$ immediately stands out as the only one for which $\langle E_{\mathrm{elst}+\mathrm{exch}}\rangle >0$. In other words, the sum of first-order interactions is net repulsive for ${\mathrm{ClO}}_{3}^{-}$ but is net attractive for each of the other anions. This observation is independent of whether one considers the bulk or interfacial data set because differences between bulk and interfacial mean values of $E_{\mathrm{elst}+\mathrm{exch}}$ are tiny in comparison to the instantaneous fluctuations, as was the case for $E_{\mathrm{int}}$. Furthermore, this anomalous prediction regarding ${\mathrm{ClO}}_{3}^{-}$ is not unique to the SAPT0 level of theory that is used in Figure 7. A similar anomaly is evident in the SAPT0(KS) results, which can been seen from the statistical distributions of $E_{\mathrm{elst}+\mathrm{exch}}$ at that level of theory that are plotted in Figure 6b. We note that the largest values of $E_{\mathrm{exch}}$ often correspond to the largest (most attractive) total interaction energies, as is seen for example in SAPT calculations of ${\mathrm{ClO}}_{n}^{-}$⋯C_6_H_6_ complexes (n=1,2,3,4) [100]. In the present case, ${\mathrm{ClO}}_{3}^{-}$ bucks this trend, according to the energy components listed in Table 2.

A possible explanation for the apparently anomalous behavior of ${\mathrm{ClO}}_{3}^{-}$ can be found by examining radial distribution functions (RDFs), g(r), obtained from the MD simulations. (These can be found in the Supporting Information for reference [7] but the salient details are described here.) Amongst the chlorine oxyanions, a unique feature of ${\mathrm{ClO}}_{3}^{-}$ is the appearance of two distinct peaks in the RDF for Cl⋯O_w_ (where O_w_ denotes water oxygen), one at r≈3.5 Å and another at r≈4.1 Å. For each of the other ${\mathrm{ClO}}_{n}^{-}$ species, the RDF consists of a single well-resolved feature at r≈3.5–3.7 Å. The shorter-*r* feature for ${\mathrm{ClO}}_{3}^{-}$ does not appear to be present in simulations based on a hybrid quantum mechanics/molecular mechanics (QM/MM) formalism, which were used to interpret x-ray scattering results [101]. If the small-*r* feature for ${\mathrm{ClO}}_{3}^{-}$ is an indication of an extraneous water molecule present at short range, then this could explain the anomalously repulsive values of $E_{\mathrm{elst}+\mathrm{exch}}$ that we then compute using quantum mechanics applied to snapshots extracted from the classical MD simulations. The presence of such a water molecule in those simulations, however, suggests that something in AMOEBA’s ion–water interaction is compensating for the short-range repulsion, or perhaps that the latter is simply not repulsive enough. Although polyvalent anions are not considered in the present work (because they are excluded from the air/water interface), it is notable that a short-*r* peak in the S⋯O_w_ RDF is also observed in our previous simulations of ${\mathrm{SO}}_{3}^{2-}$(aq) [7]. That feature is absent from QM/MM simulations and x-ray scattering experiments [102]. In view of this, AMOEBA parameterizations for both of these ions ought to be revisited. This is beyond the scope of the present work, though it is interesting to note the way that SAPT analysis of ion–water clusters was able to detect an anomaly. Notably, vertical ionization energies computed for ${\mathrm{ClO}}_{3}^{-}$(aq) and ${\mathrm{SO}}_{3}^{2-}$(aq) based on these simulations are no less accurate, as compared to experimental values [49], than what we obtain for other inorganic anions, including other ${\mathrm{ClO}}_{n}^{-}$ ions [7]. The typical accuracy is ∼0.2 eV [7], considerably smaller than the widths of the corresponding photoelectron spectra.

Returning exclusively to the monovalent ions and examining the other energy components whose statistics are summarized in Figure 7, another curiosity arises in regard to dispersion energies for the chlorine oxyanions. Dispersion is size-extensive, so that all else being equal it should scale in proportion to the number of electrons. For the ${\mathrm{ClO}}_{n}^{-}$ species, however, we observe that $|E_{\mathrm{disp}}|$ decreases in the order ${\mathrm{ClO}}_{3}^{-}>{\mathrm{ClO}}_{2}^{-}>{\mathrm{ClO}}^{-}>{\mathrm{ClO}}_{4}^{-}$. This time, perchlorate is the apparent anomaly. Dispersion energies in Figure 7c were computed using the MBD model [62], so as a sanity check, we recomputed $E_{\mathrm{disp}}$ using the third-generation *ab initio* dispersion potential *ai*D3 [50], which consists of atom–atom $C_{6}$ and $C_{8}$ potentials fitted to dispersion-only data from high-level SAPT calculations. Dispersion energies obtained for the ${\mathrm{ClO}}_{n}^{-}$(aq) species with either MBD or *ai*D3 are provided in Table 3 in the form of ensemble averages. Both models afford rather similar dispersion energies, consistent with previous tests for cases where many-body effects are not significant [62]. (In the context of dispersion, “many-body” implies an effect that cannot be described by pairwise atom–atom potentials [60,103]. Many-body dispersion effects typically arise in conjugated molecules where screening significantly modifies the effective $C_{6}$ coefficients [104]. For small molecules, three-body dispersion effects are typically quite small [90].) Notably, in the *ai*D3 model the $C_{6}$ and $C_{8}$ coefficients depend only on atomic number and do not respond to the electronic structure of the monomers.

The sharp drop in dispersion between chlorate (${\mathrm{ClO}}_{3}^{-}$) and perchlorate is a feature of both the MBD and *ai*D3 dispersion models, suggesting that this is not an artifact. A likely explanation is that, in perchlorate, the addition of a fourth oxygen atom around the central (and more polarizable) chlorine atom screens the water molecules from this polarizable center and thus significantly attenuates chlorine’s contribution to the dispersion energy. In contrast, for the other ${\mathrm{ClO}}_{n}^{-}$ ions the chlorine atom remains solvent-exposed, and the dispersion is much larger. This mechanism would be reflected in both dispersion models, if only as a function of increased chlorine–water distance in the *ai*D3 case. Also in support of this hypothesis are the data in Figure 7b for SAPT0 + MBD induction energies, which also exhibit a pronounced drop in magnitude between ${\mathrm{ClO}}_{3}^{-}$ and ${\mathrm{ClO}}_{4}^{-}$. As compared to dispersion interactions, polarization effects decay more slowly with distance, e.g., as $r^{-4}$ for charge–dipole polarization, but this dependence is still rather steep.

**Figure 8:**
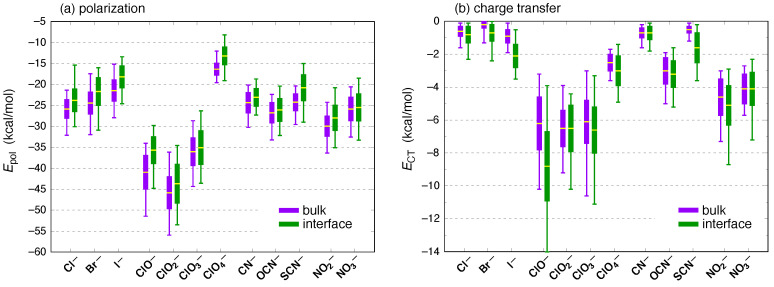
Mean values, standard deviations, and extremal values of (**a**) the CT-free polarization energy ($E_{\mathrm{pol}}$) and (**b**) the CT energy ($E_{\mathrm{CT}}$), computed at the SAPT0 + MBD/6-311+G(d,p) level. Together, $E_{\mathrm{pol}}+E_{\mathrm{CT}}=E_{\mathrm{ind}}$, and the sum (induction energy) is plotted in Figure 7b.

Polarization is often invoked in discussions of ions at the air/water interface [9,10,11,12,13,14,15,16,17], so it is interesting to note that induction energies are systematically smaller in the interfacial environment; see Figure 7b. As with the total interaction energies, however, the difference between bulk and interfacial mean values $\langle E_{\mathrm{ind}}\rangle$ is small in comparison to the instantaneous fluctuations as measured by the standard deviation. (Numerical data corresponding to Figure 7b are provided in Table 1.) Note that “polarization” as it is typically understood means strictly *intramolecular* redistribution of charge, with CT considered as a separate effect; these two parts of the induction energy are separated in Figure 8. Because the CT-free polarization energy ($E_{\mathrm{pol}}$) is much larger than the CT energy ($E_{\mathrm{CT}}$), the result is that $E_{\mathrm{pol}}$ follows essentially the same trend from ion to ion as does the total induction energy, $E_{\mathrm{ind}}$. In particular, this means that the polarization energy is systematically smaller in the interfacial environment, for each of the ions considered here. Indeed, for the canonical surface-active anions Br^−^, I^−^, ${\mathrm{ClO}}_{4}^{-}$, and SCN^−^ [19,34,35,105], the polarization energy is *significantly* smaller in the interfacial environment, by at least the standard deviation of $E_{\mathrm{pol}}$ in bulk water; see Figure 8a.

That observation, in turn, is a direct result of CT energies that are systematically larger at the interface for precisely those four surface-active anions. Statistical distributions of $E_{\mathrm{CT}}$ for all of the ions are plotted in Figure 8b. In contrast to other energy components, only for $E_{\mathrm{CT}}$ do we observe a pronounced difference between averages computed for the bulk and interfacial data sets. That said, the overall scale of the CT energies is a rather small part of either the total induction energy or the total interaction energy, with $|E_{\mathrm{CT}}|≲10$ kcal/mol except in the case of interfacial ClO^−^. (Although CT energies smaller than 10 kcal/mol do play a pivotal role in establishing the directionality of hydrogen bonds [65,81], that kind of detailed analysis of a potential energy surface is not attempted in the present work, where we are interested in ensemble-averaged properties.) For Br^−^, I^−^, ${\mathrm{ClO}}_{4}^{-}$, and SCN^−^, the average CT energy at the air/water interface is larger than its mean value in bulk water by at least one standard deviation in the bulk value. For Cl^−^(aq), the interfacial average value of $\langle E_{\mathrm{CT}}\rangle$ is larger in magnitude than the bulk value, though not quite by a full standard deviation. It is perhaps noteworthy that outliers for the CT energies tend to be larger at the interface, particularly towards negative (more stabilizing) values of $E_{\mathrm{CT}}$.

In the context of the Hofmeister series [106,107], the anions I^−^, ${\mathrm{ClO}}_{4}^{-}$, and SCN^−^ have especially large binding constants to protein [107,108], which is historically associated with the definition of chaotropes or “structure breakers” [8]. (Note that Ricci and co-workers [109] point out that the kosmotrope and chaotrope or “structure maker” and “structure breaker” labels are largely thermodynamic in origin and should not be taken too seriously in terms of their implications for microscopic hydrogen-bonding structure). In comparison to the aforementioned ions, Cl^−^ binds to proteins more weakly [108]. That said, ${\mathrm{NO}}_{3}^{-}$ is usually categorized as a structure-breaker on par with Br^−^ in the Hofmeister series [106], and as weakly surface-active on the basis of VSFG measurements [19], yet the mean values of $E_{\mathrm{CT}}$ that we obtain for ${\mathrm{NO}}_{3}^{-}$ are essentially identical in the bulk and interfacial environments, albeit with larger outliers in the interfacial case. The hypochlorite ion (ClO^−^) stands out in our analysis, with a significantly larger mean value of $|E_{\mathrm{CT}}|$ in the interfacial environment. This species is not typically discussed in the context of the Hofmeister series or in VSFG studies of the air/water interface, due to its limited stability in aqueous solution.

## 4. Conclusions

Detailed analysis of anion–water clusters extracted from MD simulations reveals that the total ion–water interaction energy (considering two solvation shells around the ion) is systematically larger for a given ion in bulk water than it is for the same ion near the air/water interface. The same is true for the CT-free polarization component of the total interaction energy, which is interesting given that polarization is often assumed to play a central role in surface activity [13], although this contention is disputed [23,24]. In any case, we observe systematically larger polarization energies in bulk water for both the “soft” anions with low surface charge density that are usually considered to be surface active (Br^−^, I^−^, ${\mathrm{ClO}}_{4}^{-}$, and SCN^−^), as well as for hard anions that are not considered to be surface active (CN^−^, OCN^−^, and ${\mathrm{NO}}_{2}^{-}$). That said, systematic differences in the mean values $\langle E_{\mathrm{int}}\rangle$ and $\langle E_{\mathrm{pol}}\rangle$ in bulk versus interfacial environments are rather small in comparison to the magnitude of the instantaneous fluctuations in $E_{\mathrm{int}}$ and $E_{\mathrm{pol}}$.

Anion-to-water CT stands out as the only energy component whose magnitude is larger at the air/water interface for some of the ions. In fact, it is larger specifically for the traditional surface-active anions: Br^−^, I^−^, ${\mathrm{ClO}}_{4}^{-}$, and SCN^−^. However, ${\mathrm{NO}}_{3}^{-}$ can also be detected in surface-sensitive vibrational spectroscopy [19], yet for that species $\langle E_{\mathrm{CT}}\rangle$ is essentially the same at the interface as it is in bulk water. The Cl^−^ ion is a borderline case whose average CT energy is slightly more stabilizing at the interface, albeit by less than one standard deviation in the fluctuations. In all cases, the CT energy constitutes less than 20% of the total induction energy, meaning that it is at least 5× smaller than the CT-free polarization energy, the latter of which does not exhibit a surface preference and is in fact larger in bulk water. Nevertheless, the consequences of this “excess” CT for soft anions at the air/water interface seem worth considering in future work, especially in the context of VSFG experiments. Intermolecular CT mechanisms have been invoked in the past to explain the surface charge of liquid water that is inferred from electrophoretic measurements [110,111,112,113].

Considering the halide ions as a series that ranges from kosmotropic to chaotropic [8], or equivalently whose surface activities decrease in the order $I^{-}>{\mathrm{Br}}^{-}>{\mathrm{Cl}}^{-}\gg F^{-}$, it has previously been noted that no single mechanistic explanation for this ordering can be gleaned from atomistic simulations [21,24]. Changes in the water–water interactions as the the anion approaches the interface appear to play a role [21]. The present analysis, based on accurate quantum-mechanical calculations of ion–water interaction energies, supports the notion that ion–water interactions alone do not readily afford any kind of a diagnostic (let alone a mechanism) to determine whether an ion resides in a bulk or interfacial environment. This null result complements our recent conclusion that short-range (first-shell) solvation structure is extremely similar in the bulk and interfacial environments [7]. The detailed mechanism of soft anion surface activity remains an open question.

## Figures and Tables

**Table 1:** Ensemble-averaged interaction energies $\langle E_{\mathrm{int}}\rangle$ and induction energies $\langle E_{\mathrm{ind}}\rangle$, computed at the SAPT0 + MBD/6-311+G(d,p) level including the *δ*HF correction for induction *^a^*.

Ion	$\langle E_{\mathrm{int}}\rangle$ (kcal/mol)			$\langle E_{\mathrm{ind}}\rangle$ (kcal/mol)	
	Bulk	Interface		Bulk	Interface
Cl^−^	−106.6 ± 8.4	−104.4 ± 9.0		−26.4 ± 2.4	−24.6 ± 2.7
Br^−^	−103.4 ± 9.6	−95.5 ± 9.9		−24.6 ± 2.7	−22.4 ± 3.3
I^−^	−94.1 ± 7.9	−87.5 ± 7.8		−22.3 ± 2.7	−20.3 ± 2.8
ClO^−^	−126.7 ± 9.1	−118.9 ± 8.7		−47.0 ± 5.1	−44.5 ± 4.3
${\mathrm{ClO}}_{2}^{-}$	−136.2 ± 8.8	−132.2 ± 9.7		−52.3 ± 4.7	−50.2 ± 5.3
${\mathrm{ClO}}_{3}^{-}$	−85.8 ± 10.0	−83.6 ± 12.5		−42.1 ± 4.1	−41.7 ± 4.4
${\mathrm{ClO}}_{4}^{-}$	−82.5 ± 7.1	−77.0 ± 9.1		−18.8 ± 1.8	−16.2 ± 2.1
${\mathrm{CN}}^{-}$	−105.6 ± 8.9	−105.4 ± 8.8		−25.0 ± 2.6	−23.8 ± 2.2
${\mathrm{OCN}}^{-}$	−110.4 ± 8.2	−110.4 ± 8.0		−29.9 ± 3.0	−29.3 ± 2.9
${\mathrm{SCN}}^{-}$	−92.2 ± 6.9	−83.5 ± 9.5		−24.6 ± 2.2	−22.4 ± 3.5
${\mathrm{NO}}_{2}^{-}$	−112.7 ± 8.3	−110.5 ± 10.3		−34.5 ± 3.2	−33.0 ± 3.5
${\mathrm{NO}}_{3}^{-}$	−106.2 ± 7.6	−106.2 ± 7.9		−29.9 ± 3.2	−29.5 ± 3.3

*^a^* Uncertainties represent one standard deviation.

**Table 2:** Ensemble-averaged energy components for ${\mathrm{ClO}}_{n}^{-}$(aq) in bulk water computed at the SAPT0 + MBD/6-311+G(d,p) level of theory.

Ion	Dipole	Energy Components (kcal/mol) *^a^*				
	Moment (D) *^b^*	$\langle E_{\mathrm{int}}\rangle$	$\langle E_{\mathrm{elst}}\rangle$	$\langle E_{\mathrm{exch}}\rangle$	$\langle E_{\mathrm{ind}}\rangle$	$\langle E_{\mathrm{disp}}\rangle$
ClO^−^	3.04	−126.7	−137.6	90.2	−47.0	−32.2
${\mathrm{ClO}}_{2}^{-}$	3.20	−136.2	−149.6	103.6	−52.3	−37.8
${\mathrm{ClO}}_{3}^{-}$	2.46	−85.8	−120.6	125.1	−42.1	−48.2
${\mathrm{ClO}}_{4}^{-}$	0.00	−82.5	−78.1	41.1	−18.8	−26.7

*^a^*$\langle E_{\mathrm{int}}\rangle =\langle E_{\mathrm{elst}}\rangle +\langle E_{\mathrm{exch}}\rangle +\langle E_{\mathrm{ind}}\rangle +\langle E_{\mathrm{disp}}\rangle$, up to roundoff error in the averaging. *^b^ω*B97X-V/6-311+G(d) level of theory at the optimized gas-phase geometry, with the center of nuclear charge as the origin.

**Table 3:** Dispersion energies for ${\mathrm{ClO}}_{n}^{-}$(aq) computed using different models and averaged over the bulk and interfacial data sets.

System		$\langle E_{\mathrm{disp}}\rangle$ (kcal/mol) *^a^*	
		*ai*D3	MBD *^b^*
ClO^−^	bulk	−28.0±2.3	−32.2±2.6
ClO^−^	interface	−24.4±2.2	−27.9±2.5
${\mathrm{ClO}}_{2}^{-}$	bulk	−35.3±2.8	−37.8±2.9
${\mathrm{ClO}}_{2}^{-}$	interface	−34.8±3.2	−37.2±3.4
${\mathrm{ClO}}_{3}^{-}$	bulk	−49.7±3.8	−48.2±3.3
${\mathrm{ClO}}_{3}^{-}$	interface	−47.5±4.4	−46.0±4.1
${\mathrm{ClO}}_{4}^{-}$	bulk	−28.4±2.6	−26.7±2.5
${\mathrm{ClO}}_{4}^{-}$	interface	−22.7±4.5	−20.9±4.2

*^a^* Uncertainties represent one standard deviation. *^b^* Based on HF monomer wave functions.

## Data Availability

The data that support this study are available from the corresponding author upon reasonable request.

## Supplementary Materials

The following are available online: AMOEBA parameter file and coordinates for each of the structures used.

## Sample Availability

Samples of the compounds are available from the authors.
